# The role of nutrition in the prevention of sarcopenia

**DOI:** 10.1016/j.ajcnut.2023.08.015

**Published:** 2023-08-30

**Authors:** Sian Robinson, Antoneta Granic, Alfonso J. Cruz-Jentoft, Avan A. Sayer

**Affiliations:** 1 AGE Research Group, Translational and Clinical Research Institute, Faculty of Medical Sciences, Newcastle University, Newcastle, United Kingdom; 2 NIHR Newcastle Biomedical Research Centre, Newcastle University and Newcastle upon Tyne Hospitals NHS Foundation Trust, Newcastle upon Tyne, United Kingdom; 3 Hospital Universitario Ramón y Cajal (IRYCIS), Madrid, Spain

**Keywords:** diet, sarcopenia, prevention, aging, lifecourse

## Abstract

Sarcopenia is a common skeletal muscle disorder characterized by a loss of muscle mass and impaired muscle function that is associated with poor health outcomes. Although nutrition is considered an important factor in the etiology of sarcopenia, the preventive potential of diet, specifically the extent to which differences in habitual patterns of diet and/or nutrient intakes impact risk of its development, is poorly understood. This narrative review considered research evidence on dietary patterns and nutrient intakes in mid- (<60 y) and young-older (60-70 y) adulthood to evaluate how they relate to age-related changes in muscle mass and function. A key finding was that current evidence on adult diet and sarcopenia risk in older age is limited and fragmented, with different outcomes reported across studies (for example, lean mass, strength) and few reporting links to incident diagnosed sarcopenia. As these outcomes are not interchangeable, it challenges collation of the evidence, leaving many gaps in understanding. There is also limited information about adult (<70 y) diet and few longitudinal studies with repeated dietary assessments to enable definition of cumulative exposures across adulthood. However, despite these limitations, findings from studies of dietary patterns already provide reasonably consistent messages about the benefits of diets of higher quality in earlier adulthood for later physical performance, although whole-diet intervention trials are urgently needed to understand their potential. In comparison, there is little evidence of benefits of higher intakes of individual nutrients in earlier adulthood for later muscle mass and function. Although these gaps need to be addressed in future research, there may already be sufficient data to promote messages about diet quality more widely - that healthier diets of higher quality across adulthood, with known benefits for a range of health outcomes, are also linked to the effective preservation of muscle mass and function.

## Introduction

Sarcopenia is a common skeletal muscle disorder that is characterized by a loss of muscle mass and impaired muscle function [1]. It is more prevalent in older age and is associated with poorer health outcomes that include an increased risk of falls, hospitalization, and mortality [2,3]. The estimated economic costs of sarcopenia, arising principally from associated clinical and informal care needs, are substantial [4,5] with further increases projected as populations age [6].

In recent decades, the wider recognition of sarcopenia as a public health concern has led to it becoming a significant area of research interest [7] and much has been learned about its multifactorial determinants, the pathophysiology of skeletal muscle loss, and underlying mechanisms. Regarding treatment, no licensed drug treatments are currently available for sarcopenia [8]. However, the benefits of resistance exercise training are well known [[9], [10], [11]]; the International Clinical Practice Guidelines for Sarcopenia (ICFSR) recommend the prescription of resistance exercise training as a principal management strategy for sarcopenia [12]. Although nutrition is considered an important influence on skeletal muscle [13], there are many gaps in current evidence that limit the translation of findings to population dietary recommendations [14]. The ICFSR guidelines therefore only make a conditional recommendation for dietary interventions to increase protein intakes as a component of sarcopenia management [12].

A notable gap in current understanding is the preventive potential of diet – specifically the extent to which differences in habitual patterns of diet and/or nutrient intake impact risk of development of sarcopenia [15] – and this potential remains underexplored. Dietary recommendations relating to sarcopenia are commonly based on consensus, with little clinical trial evidence to inform preventive strategies [14]. In this narrative review, we therefore, appraise the evidence of the effects of diet on risk of development of sarcopenia (and related components: low skeletal muscle mass, impaired muscle function, and limitations in physical performance) [16] and consider the role of dietary change as an option in primary prevention strategies to protect these components. Recognizing that skeletal muscle mass and function in later life depend both on maximal levels reached in early adulthood and their later rates of decline [17], we focused on diet and nutrition in younger adulthood (<70 y). To our knowledge, few reviews have addressed the role of habitual diet across adulthood in relation to subsequent sarcopenia risk. We limited our review to prospective observational data and intervention studies to examine how current evidence may inform opportunities to minimize or prevent age-related declines in muscle mass and function in older age and considered the implications for sarcopenia prevention.

## Why Is Nutrition Important?

**FIGURE 1:**
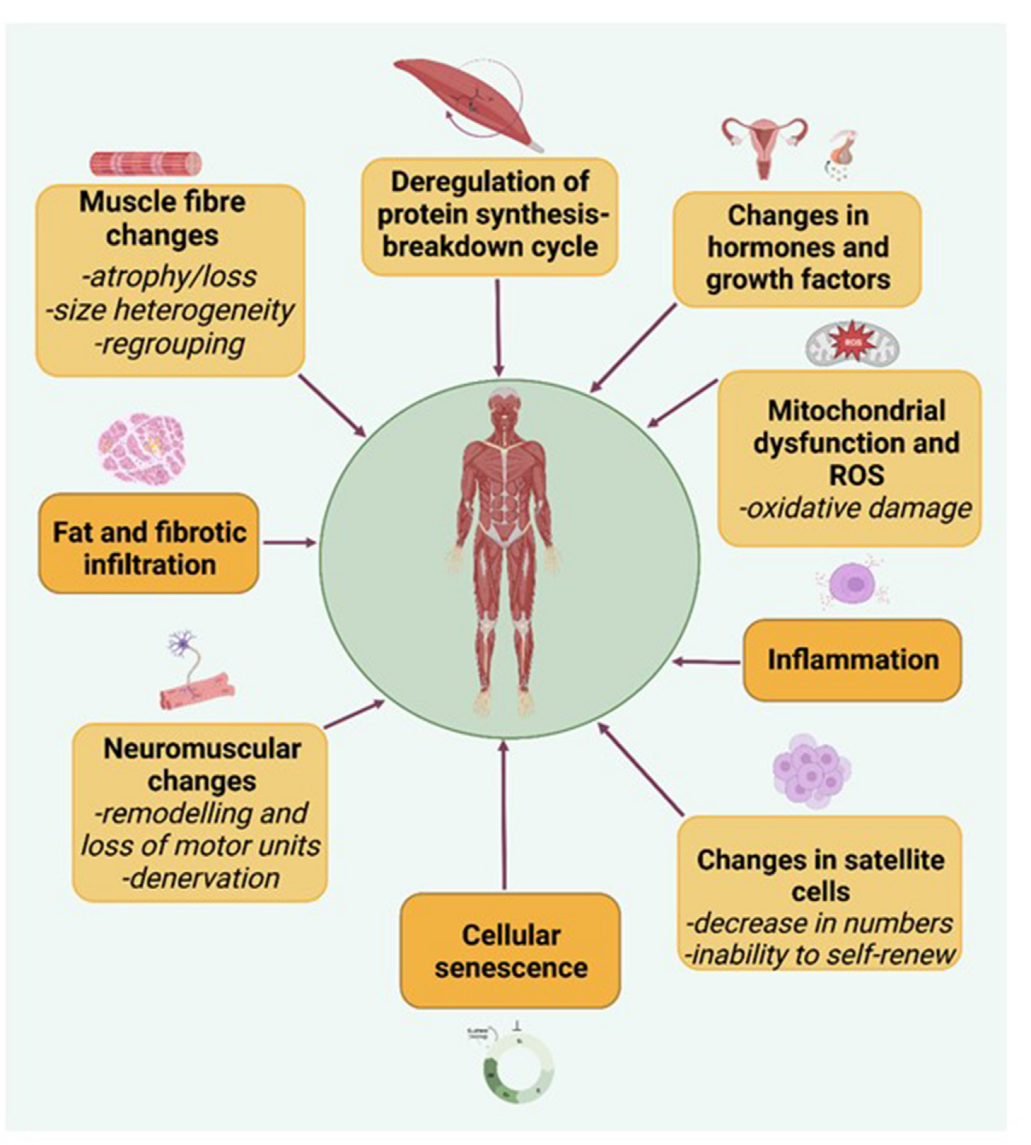
Mechanisms implicated in the pathogenesis of sarcopenia. Mechanisms of sarcopenia are complex and include skeletal muscle fiber atrophy, imbalance of muscle protein synthesis and breakdown, mitochondrial dysfunction and accumulation of ROS, and neuromuscular changes. (Created with BioRender.com). ROS, reactive oxygen species.

The maintenance of skeletal muscle mass depends on a balance of anabolic (protein synthesis) and catabolic (protein degradation) processes. Aging disturbs the homeostasis of skeletal muscle, impacting this balance and resulting in overall loss [8], occurring alongside changes in muscle composition. Age-related changes in muscle mass are significant, with estimates of an average loss of around 3%–8% per decade from mid-adulthood, increasing after the age of 60 y [18]. Declines in muscle function are more rapid. For example, longitudinal studies of older adults (aged ∼75 y) show annual losses of muscle strength of 2.5%–3% among females and 3%–4% among males [19]. There are now consensus criteria for the diagnosis of sarcopenia; it is recognized to develop both acutely and progressively and can be categorized as primary (age-related) or secondary (exacerbated by other factors such as hospitalization and illness) [20]. Although the underlying mechanisms are not fully understood, variations in habitual diets and nutritional status have implications for a number of identified multifactorial causes of sarcopenia, such as inflammation and oxidative damage, alongside other potential mechanisms that include effects on muscle protein synthesis and fat infiltration (Figure 1).

Firstly, although distinct clinically, low muscle mass is a common characteristic of malnutrition [1], as falling food consumption leads to insufficient energy intake that results in losses of muscle mass as well as body weight [21]. Even among healthy older adults, age-related falls in intake may be sizable; for example, in a comparison of healthy older adults (aged ∼70 y) with younger adults (aged ∼26 y), this amounted to a difference in energy intakes of around 16%–20% [22]. The overlap between malnutrition and sarcopenia may be significant [23,24]; for example, in a recent meta-analysis of pooled data from 7 studies of hospitalized older adults, the odds ratio (OR) for malnutrition among sarcopenic patients was 4.06 (95% CI: 2.43, 6.80) [25]. The Global Leadership Initiative on Malnutrition (GLIM) proposes low skeletal muscle mass as 1 of 5 consensus criteria for the diagnosis of malnutrition, with a recommendation to promote the wider assessment of muscle mass as an integral component of the GLIM diagnosis [1,26].

Secondly, less extreme nutrient insufficiency also has important implications for several mechanisms underlying muscle loss. The macronutrient most extensively researched in relation to sarcopenia is protein, as it is an anabolic stimulus that increases muscle protein synthesis after feeding, as well as a source of amino acids [27]. There is some evidence that anabolic responses to feeding are blunted among older adults, suggesting that higher protein intakes are needed in older age to preserve muscle mass and function [28], particularly in combination with exercise training [29]. However, the role of differences in habitual diets, and whether protein intakes below recommended levels in older age contribute to imbalances in protein metabolism and accelerated losses of skeletal muscle mass and function, continue to be topics of debate [15,28].

Low intakes of a range of other nutrients and dietary constituents are also implicated in the etiology of sarcopenia. Particularly, significant research effort has been directed toward understanding the effects of vitamin D insufficiency, potentially mediated by the vitamin D receptor and possible anti-inflammatory actions [27,30]. Other dietary constituents of interest may impact inflammatory processes and oxidative stress. These include n-3 fatty acids that have known anti-inflammatory actions [31], as well as possible direct effects on muscle protein synthesis [32], and dietary antioxidants such as carotenoids, selenium, and polyphenols that can help counter the effects of reactive oxygen species (ROS) that cause damage to biomolecules in muscle and, via signaling pathways, are linked to inflammation [33]. As a major source of ROS generation, muscle mitochondria are targets for oxidative damage, with increasing oxidative stress promoting mitochondrial dysfunction and deterioration in muscle homeostasis [34].

More recently, in a move away from the examination of single nutrients, the importance of whole-diet effects is increasingly recognized. There is a growing evidence base that links higher diet quality and nutrient-dense dietary patterns that provide greater amounts of a range of bioactive nutrients and phytochemicals, as well as a favorable acid-base balance, to beneficial effects on muscle mass and function [35]. For example, consideration of the overall inflammatory potential of the diet, which accounts for the balance of pro- and anti-inflammatory constituents, may help improve understanding of the impact of the whole diet on inflammatory processes underlying muscle loss [36,37]. Besides impacting nutrient intake, such qualitative differences in diet can affect the composition of the gut microbiota [38], with the potential to impact skeletal muscle mass and function via effects on inflammatory processes and anabolic resistance [39].

Consistent with potential mechanistic links to nutrient insufficiency, a number of studies of the diets of sarcopenic older adults describe lower energy and nutrient intakes when compared with nonsarcopenic adults. For example, in the first systematic review and meta-analysis of these studies (published between 2006 and 2019) a mean difference in daily energy intake of -157 kcal (95% CI: -195, -118) was shown between sarcopenic older adults (19 studies) and a mean difference in daily protein intake of -5.6 g (95% CI: -7.9, -3.2g) (12 studies) when compared with their nonsarcopenic counterparts [40]. An important limitation of this evidence is that most of the studies comparing intakes of sarcopenic compared with healthy older adults in this review were cross-sectional. The possibility of reverse causality therefore cannot be excluded, such that losses of muscle strength and impairments in physical performance result in changed eating behavior and lower nutrient intake, rather than poor diet causing these outcomes [41,42].

This has also been a limitation of much of the cohort evidence to date, limiting the causal inferences that can be drawn to further understand the role and importance of diet as an influence on age-related losses of muscle mass and function. Although there is now good trial evidence showing that nutritional supplementation is an effective intervention for the treatment and management of sarcopenia, particularly when combined with exercise training [43,44], the implications of these findings for nonsarcopenic older adults and their potential extrapolation to dietary guidance for healthy older populations to delay muscle loss and prevent sarcopenia need to be established.

## Diet in Adult Life and Sarcopenia Risk

This review considers evidence on diets in mid-adulthood (<60 y) and young-older adulthood (60–70 y) in relation to later risk of sarcopenia and/or decline in its components (muscle mass, function, physical performance). Our search strategy included terms that described an aspect of diet (such as “dietary patterns,” “diet quality,” “Mediterranean,” “protein,” “fruit,” “vegetable,” “vitamin,” “antioxidant”) combined with the following terms: “longitudinal,” “cohort,” “prospective,” “incident,” “trial,” “intervention.” The key outcome terms were as follows: “sarcopenia,” "muscle weakness," "muscle atrophy," "muscle strength," "muscle mass," "physical performance," "physical function." The search was supplemented by studies known to the authors, along with other forward citation searches. Much of the published evidence from studies of participants aged <70 y, as set out in the following sections, comes from prospective observational studies; for many dietary exposures, there is currently little trial evidence on diet alone; many interventions combine dietary changes with exercise training. This review focused on diet, particularly to understand the role of habitual diet in the etiology of sarcopenia. We excluded cross-sectional studies and dietary interventions, such as energy restriction, which may lead to a loss of muscle mass. We did not consider specific interventions to prevent sarcopenic obesity (low muscle mass and high-fat mass) [45].

### Dietary patterns

A growing number of observational studies have considered the role of the whole diet in the etiology of sarcopenia, enabling the synthesis of this evidence in recent systematic reviews [[46], [47], [48], [49], [50], [51], [52], [53], [54]] and a meta-analysis of its findings [52]. There are many challenges in collating the evidence, arising from significant differences in study methodology and design and, particularly, in the description of dietary patterns. For example, in the recent systematic review and meta-analysis by Van Elswyk et al. [52], which included 37 studies, 26 methods were used to describe dietary patterns, including a priori scoring approaches as well as a posteriori data-driven methods. In their review, the largest group of studies characterized participants’ adherence to a Mediterranean-dietary pattern (6 scoring methods); other studies evaluated diet quality defined using different scoring methods, including Healthy Diet Indicators, the Baltic Sea Diet, and the Dietary Quality Index-International; 5 studies reported patterns defined using factor/cluster analysis or macronutrient distribution.

In terms of understanding causal links between diet quality and sarcopenia risk, as well as the potential of habitual diet to influence declines in muscle mass and function in older age, evidence is limited. Many whole-diet studies are of older populations (>70 y), with much less evidence for younger adults. Furthermore, studies differ in design with respect to muscle outcomes evaluated, duration of follow-up, timing of dietary assessment, consideration of sex differences, as well as the definitions of dietary patterns, all of which challenge the simple collation of this evidence. However, while reviews often consider studies according to the type of dietary pattern described, many patterns share common tenets, such as high consumption of fruits, vegetables, and wholegrain cereals, and lower consumption of highly processed foods (or the reverse for unhealthy patterns). Given the limited number of studies, we therefore group dietary patterns, indicating variations in the overall diet quality. Importantly, the recent meta-analysis by Van Elswyk et al. [52] found low statistical heterogeneity among studies that had used different methods of dietary pattern analysis, providing some support for this approach.

#### Mid-adulthood

Prospective studies have considered participants’ cumulative exposure to dietary patterns across mid-adulthood in different ways. Although dietary patterns are expected to track over time, with stability in the ranking of individuals within the population, absolute dietary pattern scores may change [55]. Studies with a single dietary assessment at baseline may therefore be less informative than those that can take account of repeat dietary assessments over time, or those using estimates of average exposure across several assessments, to evaluate in relation to subsequent measures of muscle mass and function. To date, few studies have described dietary patterns assessed from repeated measures of diet across mid-adulthood, and outcomes differ across these studies. However, overall, they provide some indication of the benefits of higher quality diets in middle adulthood in relation to muscle mass and function 10–20 y later. For example, in the Geelong Osteoporosis Study, Davis et al. [56] reported that a more pro-inflammatory diet at baseline (age 50 y) was associated with a greater decrease in the skeletal muscle mass index (SMI) among 522 males when followed up at 15 y, as well as a slower time on the timed up-and-go (TUG) test; higher scores for a traditional diet were associated with greater decreases in SMI but not with measured function at follow-up. Comparable associations with SMI have also been reported for females in this cohort [57].

In contrast, higher quality diets in mid-adulthood have been linked to better functional outcomes in later life in other cohorts [58,59], although the functional measures differed across studies. A key part of the evidence on diet in mid-adulthood has come from the Nurses’ Health Study (*n* = 54,762 females) [42], in which risk of self-reported functional impairment was assessed (36-item short-form survey (SF-36) scores ≤80) every 4 y from 1992 (age ∼55 y) to 2008, in relation to cumulative average diet scores [the Alternative Healthy Eating Index-2010 (AHEI-2010); mean of all scores from 1980 to the start of each follow-up period]. Over an 18-y follow-up period, greater adherence to AHEI-2010, indicating a healthier diet, was shown to be associated with a lower risk of incident physical impairment [multivariable-adjusted hazard ratio (HR), comparing participants in the highest and lowest quintiles of AHEI-2010 scores = 0.87 95% CI: 0.84, 0.90]. Comparable indications of the benefits of a healthier dietary pattern across mid-adulthood, for longer-term physical performance, were also observed in a study of 969 males and females in the MRC National Survey of Health and Development (NSHD) in the United Kingdom, in which diet was assessed at ages 36, 43, 53 and 60–64 y. Higher diet quality scores at each age and an overall measure of diet quality across adulthood were associated with better measured physical performance (chair rise speed, standing balance time, TUG speed) at 60–64 y; these associations were largely robust to adjustment (factors including age at assessment, physical activity, smoking history) [55]. However, the strongest associations were observed using contemporary dietary data in the cross-sectional analyses at 60–64 y, with conditional models also providing evidence that changes in diet to increase diet quality up to this age (relative to younger ages) were associated with better measures of function in 2 tests (chair rise speed, standing balance time). This encouraging finding could be key to informing the timing of dietary strategies to protect physical performance in older age, although further data are needed.

#### Young-older adulthood

Compared with mid-adulthood, there is relatively more evidence on the dietary patterns of young-older adults (aged 60–70 y) in relation to later physical outcomes, although generally the studies are of shorter duration (3–10 y). Like studies of younger adults, outcome measures differ markedly across studies and mainly include functional outcomes (self-reported as well as measured). Overall, a number of studies suggest protective effects of higher quality diets [[60], [61], [62], [63], [64]], particularly for physical performance [52], although the different study designs, populations, and outcomes limit opportunities for simple collation of this evidence. Some studies indicate sex differences in diet quality effects, although this is not always a consistent finding. For example, Perala et al. [65,66] evaluated associations between compliance with a Nordic diet (characterized by greater consumption of fruits and berries, vegetables, and fish) among 600 females in the Helsinki Birth Cohort at baseline (age ∼61 y), in relation to measured muscle strength and physical performance (Senior Fitness Test) at the 10-y follow-up. In females, greater compliance with the Nordic pattern was associated with higher muscle strength (hand grip, knee extension) and better physical performance, but no associations were observed among males. Similarly, in a further follow-up of the NSHD cohort, higher diet quality (Healthy Eating Index-2015) at 60–64 y was associated with faster walking speed in females aged 69–71 y, although no differences were observed in males [67]. However, other studies report similar findings for both males and females [41], whereas, in some populations, the beneficial effects of higher quality diets have been reported among males but not females [68].

The recently published data by Talegawkar et al. [41], from a study of 1358 males and females in the Baltimore Longitudinal Study of Aging, are an important addition to the evidence base. The study is notable in several respects, including the repeated assessments of diet, long duration of follow-up, and comprehensive measures of physical performance. Participants' mean Mediterranean-Dietary Approaches to Stop Hypertension Intervention for Neurodegenerative Delay (MIND) scores were calculated from repeat dietary assessments; the mean age at the first diet visit was 68 ± 14 y. Those who had higher mean MIND scores had lower odds of physical function impairment [Short Physical Performance Battery (SPPB) score <10] at follow-up (median 6 y, range 0–13 y). For example, participants with mean MIND scores in the top third of the distribution had 46% lower odds of impairment in repeated chair stands when compared with the bottom; the equivalent figures were 51% for standing balance and 49% for gait speed. Importantly, higher MIND scores were also associated with a greater grip strength at follow-up in both males and females aged >60 y, amounting to 1.86 kg (95% CI: 0.33, 3.40) comparing males in the top and bottom thirds of scores, and 1.24 kg (95% CI: 0.04, 2.45) among females (fully-adjusted models).

**FIGURE 2:**
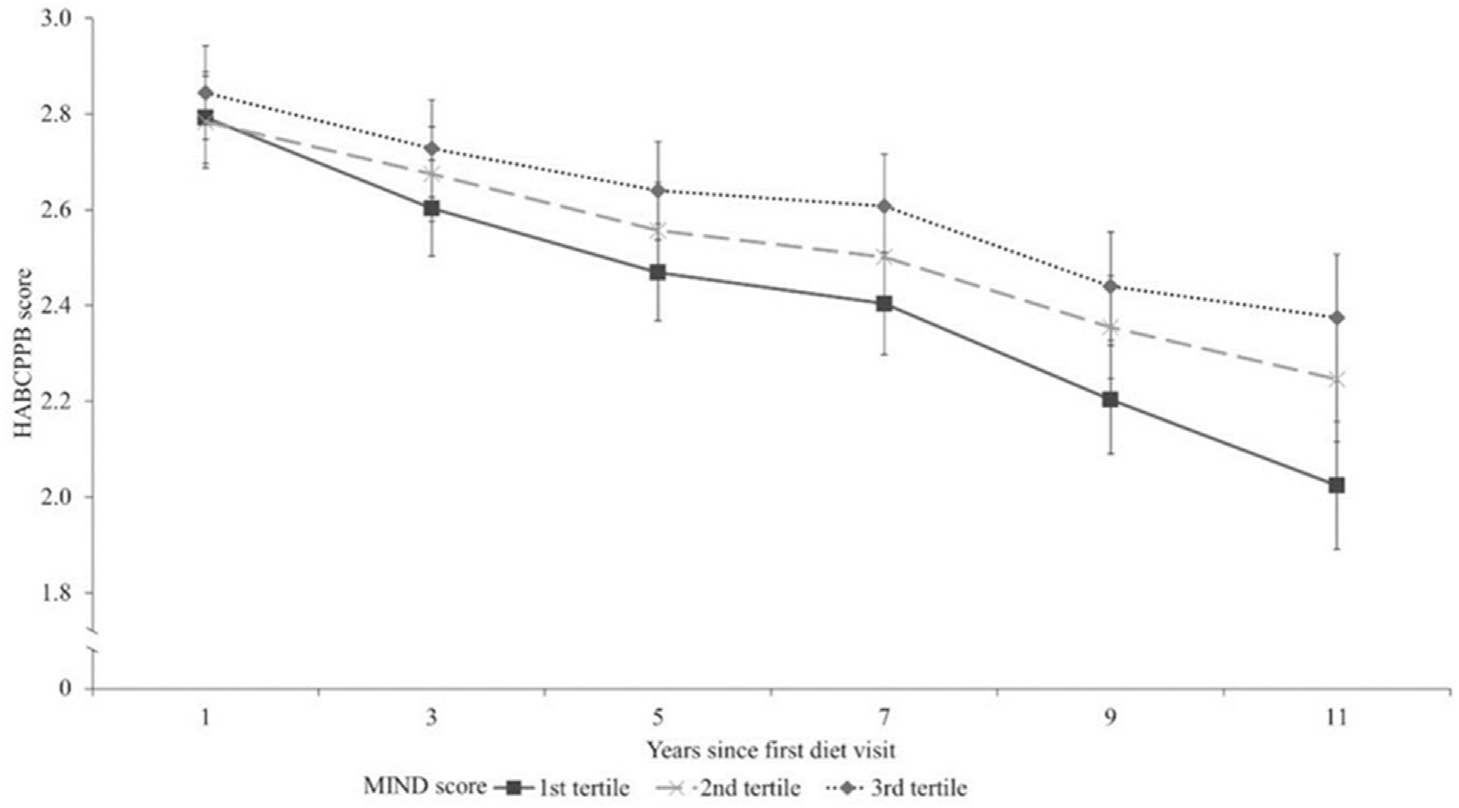
HABCPPB decline over follow-up time, stratified by the MIND diet score tertile, adjusting for age, race, years since first diet visit, years of education, smoking status, physical activity, number of chronic diseases, BMI, and mean energy intake. Reproduced with permission from Talegawkar et al. [41]. HAPBCPPB, health, aging, and body composition physical performance battery; MIND, Mediterranean-dietary approaches to stop hypertension Intervention for Neurodegenerative Delay.

In this study, functional decline was assessed with the Health, Aging, and Body Composition Physical Performance Battery (HABCPPB). Higher MIND scores were associated with slower functional decline - seen among participants with scores in the upper two-thirds of distribution when compared with those in the bottom third (Figure 2).

There are some key findings from this study of diet at young-older age, highlighted by their consistency (comparable associations in males and females, consistent evidence of beneficial effects for measured physical performance and strength) and large effect sizes, which point to the importance of this profile of diet (characterized by a more frequent consumption of vegetables, berries, nuts, and whole grains, and less frequent consumption of red meat and meat products, fast-fried foods, pastries, and sweets) [41].

There are few whole-diet trials with measures of muscle mass and function as primary outcomes. For example, while the most sizable literature on dietary patterns is for the Mediterranean pattern, a recent systematic review of this evidence did not identify any clinical trials [69]. However, there is limited intervention evidence suggesting that improvement in diet quality in young-older adulthood when combined with exercise training has protective effects [70,71].

#### Summary

Studies with repeated assessments of diet across adulthood suggest that habitual diets of higher quality are protective and linked to better-measured physical performance at later follow-up. Although differences in study design limit direct comparison, overall messages across studies are generally positive. The current promotion of plant-based diets as part of sustainable dietary guidelines should lead to diets of higher quality that will have beneficial effects. However, the evidence is incomplete, with little information on muscle strength and even less information on incident diagnosed sarcopenia, and the potential for whole-diet interventions to improve diet quality with or without exercise training remains to be explored. Additionally, while there are concerns about the complete exclusion of animal-based foods for older vegan consumers, little is known about the impact of veganism on skeletal muscle mass and function [72]. Further data are needed, particularly from intervention studies in healthy adult populations, to inform prevention opportunities.

### Foods

Alongside the investigation of the effects of the overall quality of diet, there is growing interest in foods and their potential as protective or harmful influences on muscle function. Recent systematic reviews provide some support for the beneficial effects of specific foods [[73], [74], [75]], although much of the current evidence is from studies of older adults and/or is cross-sectional in design.

#### Mid-adulthood

The foods linked most consistently to beneficial effects on muscle function across studies of middle-aged adults are fruits and vegetables. Some information comes from dietary patterns studies, in which score components have been considered separately from overall diet scores. For example, in the Nurses’ Health Study, Hagan et al. [42] reported that the strongest relationships between high consumption of individual foods and a lower risk of incident physical impairment were found for fruits (oranges, orange juice, apples, pears), vegetables (romaine or leaf lettuce), and walnuts. However, these associations were modest, with the authors suggesting that the overall dietary pattern may be more important than its components in influencing subsequent function. Furthermore, protective benefits of greater consumption of fruits and vegetables are not always found. In a follow-up of an African American cohort (432 males and females; mean age 59.2 ± 4.4 y at baseline) with low fruit and vegetable consumption, higher intakes of vegetables (besides carrots, salads, and potatoes) were associated with greater grip strength at 6-y follow-up, whereas fruit juice was associated with lower grip strength [76]. Conversely, in the Atherosclerosis Risk in Communities Study [77], greater consumption of fruit at baseline (9404 males and females; mean age 53.6 y, range 44–66 y) was associated with lower odds of impaired lower-extremity function (self-reported) after ∼9 y, but associations with vegetable consumption were not seen in the fully-adjusted models.

Clearer evidence of the harmful effects of low habitual fruit and vegetable consumption in mid-adulthood may be provided by the longitudinal study of 5671 males and females Whitehall II Study in the United Kingdom [78]. Fruit and vegetable consumption was reported on 3 occasions: 17 y (mean age 49.1 ± 5.9 y), 10 y (mean age 55.4 ± 5.9 y), and 5 y (mean age 60.9 ± 5.9 y) before the assessment of walking speed and grip strength (mean age 65.9 ±5.9 y). Low consumption was defined as eating fruits and vegetables less than twice per day; a cumulative score representing the number of times low consumption was reported over the 3 assessments was calculated. Low fruit and vegetable consumption at every assessment through mid-adulthood was associated with slower walking speed at follow-up (adjusted for sociodemographic characteristics), with a suggestion that the accumulation-of-risk model fitted the data better than models with single assessments, indicating greater effects with a longer duration of low consumption. Importantly, the cumulative association was independent of other aspects of unhealthy lifestyles, including low physical activity. The associations between low fruit and vegetable consumption and grip strength also suggested an accumulation of risk over mid-adulthood, although these were weaker and not robust to adjustment for other lifestyle factors [78].

The need to meet protein requirements to prevent losses of muscle mass and function has focused attention on foods of animal origin, particularly dairy foods and meat. However, available evidence is limited and messages from different studies regarding the potential benefits of the consumption of animal foods in mid-life are mixed. For example, a study of the Framingham Offspring Cohort (2349 males and females; median age 52 y) over 9 y showed that, independently of participants’ physical activity levels, a greater consumption of animal protein-source foods (red meats, poultry, fish, and dairy) was associated with greater skeletal muscle mass percentage at follow-up [79]. However, in the Nurses’ Health Study, a greater consumption of red and processed meats was associated with a higher HR for incident physical impairment in the age-adjusted model, although there was no association in the fully-adjusted model [42]. Evidence for dairy foods is also inconclusive. For example, in the Atherosclerosis Risk in Communities Study, Houston et al. [77] reported some associations between greater consumption of dairy foods (milk, yogurt, ice cream, cheese) at baseline and lower odds of functional limitations and disability [impaired activities of daily living (ADLs) and instrumental ADLs, lower-extremity function] but the pattern of associations was not consistent for African American and White participants or for males and females. Most recently, further analysis of milk consumption in the NSHD cohort showed that a higher grand mean of total milk intake (calculated from repeat assessments in mid-adulthood) was associated with higher grip strength in males at age 53 y, but it was not related to a decline in strength in later life (69 y). Furthermore, no associations were observed among females [80].

#### Young-older adulthood

Greater fruit and vegetable consumption has also been linked to some benefits for muscle function in studies of young-older adults. However, like studies of younger populations, there is limited evidence with different outcomes considered across studies and inconsistencies in findings. For example, in the follow-up of the Helsinki Cohort Study, females’ physical performance (Senior Fitness Test) scores were positively associated with the consumption of fruits and berries, although there were no differences in muscle strength [65,66]. Similarly, in a pooled analysis of data from 3 independent cohorts (mean ages 68.7 ±6.4 y, 81.8 ±4.1y, and 74.5 ±5.8 y), there was an inverse dose-response relationship between fruit consumption at baseline and risk of slow walking speed at follow-up (mean 2.5 y), but no association with vegetable consumption, and there were no associations between fruit or vegetable consumption with muscle strength [81]. In a study of 1630 males and females in the Seniors-ENRICA cohort (age ∼68 y), Struijk et al. [63] also report an inverse association between fruit consumption, but not vegetable consumption at baseline, and risk of impairment in self-reported function (≥5-point decrease in the physical component of the 12-item short-form health survey, SF-12) at follow-up (median 3.5 y).

The links between the consumption of foods of animal origin in this age group and later muscle outcomes are also unclear. In a recent analysis of data from the EpiDoC cohort (2860 males and females), the frequency of consumption of animal foods (meat, fish, dairy products) at baseline [median age 66.6 y, interquartile range (IQR) 59.2–74.7 y] was not related to self-reported mobility limitations (difficulty standing up from a chair, walking, climbing stairs) at the 2.7-y follow-up [82]. However, the type of animal food may be important, specifically with effects differing for processed and unprocessed meat [83]. For example, in further analyses from the Seniors-ENRICA cohort, higher consumption of processed meat was associated with a higher risk of impaired agility (Rosow and Breslau scale) and lower-extremity function (SPPB) at follow-up (median 5.2 y), whereas, there were no associations with the consumption of red meat or poultry [84].

Evidence on dairy food consumption in young-older age is also limited and findings are inconsistent. For example, in a mean 3.5-y follow-up of participants in the Study on Nutrition and Cardiovascular Risk in Spain, Lana et al. [85] report that a high consumption of low-fat milk and yogurt at baseline (1871 males and females; aged ∼68 y) was associated with a lower risk of slow walking speed, but no associations were found with whole milk, yogurt, or cheese; there were no associations with muscle weakness (hand grip strength). Additionally, important evidence comes from an 18-mo intervention study of males (age ∼61 y) in which fortified milk was provided (400 ml/d) in 1 arm of the trial. In this study, milk supplementation (without exercise) had no effect on muscle function or size [86].

#### Summary

Evidence of the potential effects of differences in the consumption of individual foods in younger adulthood on muscle mass and function in older age is limited. Overall indications regarding the potential protective benefits of foods are mixed and the evidence is less consistent than that for diet quality. However, a particular challenge in understanding the observational data on individual foods is the collinearity of foods in the diet, such that the high consumption of one food may indicate a higher or lower consumption of others, rather than specific foods being causally linked to differences in muscle outcomes. These need to be adjusted for statistical models to understand independent effects. For example, findings from the Whitehall II study indicate the role of habitual low fruit and vegetable consumption, with a potentially important message regarding an accumulation of risk with longer duration across mid-adulthood [78]. However, it is also possible that low consumption of fruit and vegetables acts as a marker of differences in the wider pattern of eating, including high consumption of other foods, which may have negative effects on muscle. In this regard, the role of highly processed foods may be particularly important, as greater consumption of these foods is also a characteristic of low quality diets alongside low fruit and vegetable consumption. In a recent analysis of data from Tianjin Chronic Low-grade Systemic Inflammation and Health study, Zhang et al. [87] showed that high consumption of ultra-processed foods (UPF) in mid-adulthood (5409 males and females; median age 48.3 y) was associated with a greater annual decline in grip strength (median follow-up 3.0 y). The association was robust to adjustment for a range of factors, including other aspects of diet and baseline grip strength, and evident in subgroup and sensitivity analyses. This novel finding suggests a direct effect of UPF on muscle function, potentially via effects on low-grade inflammation [88] and/or through increased exposure to constituents that are linked to food processing, such as advanced glycation end products [89]. As diet quality captures high and low consumption of different foods, it provides a more complete assessment of dietary exposure, potentially also contributing to greater consistency across the evidence for dietary patterns than for individual foods.

### Protein

The most intensively researched macronutrient in relation to muscle mass and function is protein. Although mechanistic links to inflammation have also focused attention on the role of n-3 fatty acids [90,91], the effects of improving n-3 status are uncertain; a systematic review of supplementation studies of healthy older adults showed benefit for only 1 functional outcome [92]. Some evidence suggests that differences in macronutrient balance may be important, as diets of lower carbohydrate density and higher fat density have been linked to a greater risk of mobility limitations among older adults [93]. But, overall, current evidence and understanding of other macronutrients is limited.

The body of evidence on protein has been summarized in a number of recent systematic reviews [29,[94], [95], [96], [97], [98], [99]]. Much of the evidence comes from intervention studies, often in combination with resistance exercise training. There are fewer prospective studies that address the role of low habitual intakes of protein in relation to risk of sarcopenia.

#### Mid-adulthood

There are some observational data on protein intakes in younger adult populations but findings in relation to muscle outcomes are mixed. For example, in a follow-up of 2986 males and females in the Framingham Third Generation Study (mean age 40 ±9 y), lower protein intake [assessed in 2002–2005; 82% met the recommended daily allowance (0.8g/kg/d)] was associated with lower appendicular lean mass and quadriceps strength (measured in 2008–2011), when comparing the lowest and highest quartiles of intake [100]. In contrast, in other analyses from the NSHD cohort, there were no differences in grip strength measured at age 53 y in relation to protein intakes at earlier ages (36 y and 43 y) in adjusted models [101]. Most recently, the incidence of low grip strength was evaluated in a 4-y follow-up of 32,458 participants in the Korean Genome and Epidemiology Study (median ages: males 56 y (IQR, 49–62 y), females 53 y (IQR, 47–58 y)). There was no association between risk of incident low grip strength and protein intake at baseline, or with a change in the amount of protein over the follow-up period [102].

Many intervention trials have investigated the effects of consuming additional protein, although study designs vary considerably [29]. In their systematic review and meta-analysis of protein supplementation studies in healthy adults, Wirth et al. [96] pooled data according to age group. Among younger adults (mean age 18–55 y), supplementation resulted in gains in lean body mass (LBM), but there was no difference in muscle (leg press) strength. In this review, all intervention studies of adults in this age range were with combined exercise training. This is important, as systematic and meta-analyses published since confirm that the effects of protein supplementation on LBM are evident when combined with exercise training, but highlighting that there is a lack of data on the effects of protein supplementation alone [29,103]. There is some evidence of small gains in lower body muscle strength among adults aged <65 y, when combined with resistance exercise training [29].

#### Young-older adulthood

The most recent systematic review of observational evidence included 22 cross-sectional and 9 prospective studies. Most studies were of older adults (aged >70 y) and outside the scope of this review but, consistent with the findings for younger populations, meta-analysis of these longitudinal studies showed no association between protein intakes and longitudinal changes in functional outcomes, nor provided indication that high intakes were protective in terms of rate of decline in isometric hand grip strength or walking speed [94]. In comparison, many intervention studies have been conducted, mostly combined with exercise training [29]. Although there are some differences in the conclusions of the systematic reviews of this evidence depending on the inclusion criteria and study selection, most show that protein supplementation in combination with exercise training leads to small increases in lean mass. However, the limited number of trials on protein supplementation alone (without exercise) do not provide evidence of benefits of increasing protein intake [29]. A few systematic reviews indicate benefits of protein supplementation for functional outcomes, but only when combined with exercise training. For example, in the systematic review and meta-analysis of randomized controlled trials (RCTs) conducted with healthy adults (nonobese, nonsarcopenic), Nunes et al. [29] showed small effects on lower body strength in adults aged ≥65 y in trials of protein supplementation combined with resistance exercise training, although these were not evident in sensitivity analyses.

#### Summary

Despite widely held perceptions that older adults need to increase protein intakes to meet their greater needs, there is currently little evidence that links low habitual intakes of protein in mid-adulthood or young-older age to greater age-related declines in muscle function – or trials showing benefits of consuming additional protein. Although older sarcopenic adults living with frailty and/or malnutrition may have changed protein requirements, it is unclear from current evidence whether healthy adult populations need to increase protein intake. Nishimura et al. [28] argue that recommendations to increase protein intakes are extrapolated from the findings of acute feeding studies and that there are potential limitations to using these data to set whole-body protein requirements, which could have important implications for policymakers setting population recommendations. The recent systematic review by Hengeveld et al. [104] is therefore key, as it looked specifically at the health effects of increasing habitual protein intakes above the population reference intake (≥0.8g protein/kg/d). They found ‘insufficiently convincing data’ that increasing protein intake in older adults above the reference intake affects health outcomes.

### Micronutrients

Several mechanistic pathways point to the potential importance of micronutrients in the etiology of sarcopenia. There is a significant body of research on vitamin D, although currently much less is known about the role of other vitamins, minerals, and trace elements [105].

#### Vitamin D

Much of the evidence on vitamin D is from intervention trials, with less observational data from prospective studies to inform the present review to assess whether lower status is linked to greater age-related declines in muscle mass and function. Although there is some prospective evidence from studies of older adults, which shows that lower vitamin D status is associated with poorer functional outcomes [106,107], there is currently a lack of evidence for younger adults. As low vitamin D status is common in many populations and can be improved with supplementation, the large number of intervention studies that have been conducted to assess efficacy should be informative [108]. However, depending on the included studies, systematic reviews are inconsistent in their conclusions. For example, recent systematic reviews report positive [109,110] and negative [111] effects of vitamin D supplementation. Importantly, in a systematic review and meta-analysis of 10 RCTs published by Prokopidis et al. [111], which considered the effects of vitamin D supplementation in community-dwelling adults (>50 y) and highly relevant to this review, there was no improvement in muscle mass or strength. Furthermore, it is a concern that meta-analysis of the physical performance data (SPPB) indicated that scores decreased in the supplemented groups.

#### Antioxidant nutrients

Early prospective evidence from older populations indicated that lower antioxidant (carotenoids, vitamin E, vitamin C) intakes and status were associated with later functional decline [105]. However, there is little prospective observational evidence from mid-adulthood or young-older age to evaluate antioxidant effects, and there are very few intervention trials [74]. The findings from a 12-y follow-up of the Framingham Offspring Cohort (mean age 61± 9 y at baseline), with repeat assessments of diet and physical performance, therefore make a key contribution to current understanding [112]. In fully-adjusted models, higher averaged intakes of total carotenoids, lycopene, and lutein + zeaxanthin were associated with a smaller loss in grip strength over follow-up (*n* = 2452), while higher intakes of total and all individual carotenoids and vitamin E were associated with smaller loss in gait speed (*n* = 2422). There were no associations with vitamin C intake. However, while these findings indicate modest protective effects consistent with earlier studies in older adults, they were not confirmed in the authors’ analyses of data from a comparison cohort [112]. There are few trials of antioxidant supplementation and, to date, findings have been inconsistent [113,114]. Evidence for the effects of selenium intake and status in mid-adulthood and young-older age is also lacking from observational studies. There is little trial evidence, with a recent study showing that selenium supplementation of postmenopausal females over 6 mo did not change their grip strength or physical performance (SPPB) [115].

#### Minerals

Compared with other nutrients, much less attention has been focused on the role of minerals and their influence on muscle mass and function. The first systematic review of this evidence, published in 2018, included 10 studies examining 5 individual minerals [116]. These included only 1 prospective study and 1 intervention trial, both on older adults (>70 y). The limited evidence from younger adults includes follow-up of the Tasmanian Older Adult Cohort study, in which higher intakes of magnesium, phosphorous, and zinc at baseline (mean age 62±7 y) were positively associated with dual-energy X-ray absorptiometry-assessed appendicular lean mass over 2.6 y, although there were no associations with muscle (leg) strength [117]. Data published more recently from the Seniors-ENRICA cohort indicate the beneficial effects of higher intakes of zinc, magnesium, and potassium for functional outcomes [[118], [119], [120]], whereas the potential importance of low calcium intakes as a determinant of muscle loss over 10 y was reported in a prospective study of Korean females (aged >50 y) [121]. Clearly, there are many gaps in this evidence and further data are needed.

## Discussion

**FIGURE 3:**
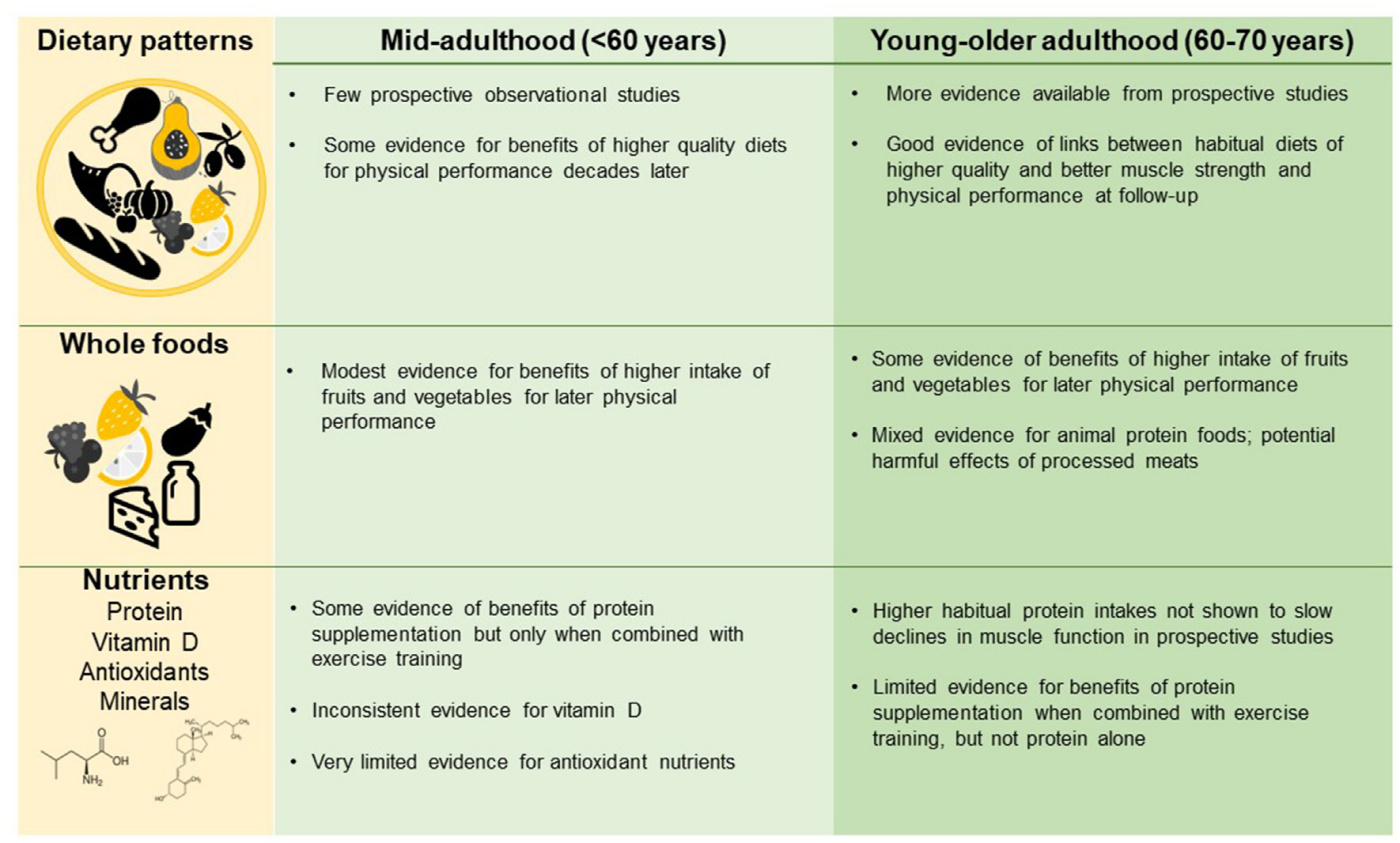
Summary of evidence from prospective studies and intervention trials for the effects of nutrition in mid- and young-older adulthood (<70 y) on later muscle mass, function, and physical performance.

This review considered evidence on the dietary patterns and nutrient intakes of younger adults (<70 y) to evaluate how differences in adult diet relate to age-related changes in muscle outcomes related to sarcopenia (muscle mass and function, physical performance). An overall summary of this evidence is shown in Figure 3.

We aimed to collate findings from observational and intervention studies to examine the preventive potential of diet in adulthood and explore the extent to which differences in habitual diet may slow or prevent declines in muscle mass and function in older age, which have implications for sarcopenia risk.

There are several key findings. First, current evidence on adult diet and sarcopenia risk in older age is limited and fragmented. An important limitation of the evidence is the marked variation in physical outcome measures used across research studies. The majority of studies report data on physical performance or risk of impairments (measured and self-reported), fewer studies include information on muscle strength or other muscle characteristics, and very few studies report links to diagnosed sarcopenia. These different indicators of muscle function cannot be considered interchangeably, limiting the collation of the evidence and leaving many gaps in understanding. Second, there is relatively less information about adult diet (compared with diet in older age) in relation to sarcopenia and its components. Much of the dietary evidence is cross-sectional, with fewer longitudinal studies that include repeated dietary assessments across adulthood to enable definition of individuals’ cumulative exposures over time, needed to inform the preventive potential of diet and/or identify protective aspects, and there are few relevant trial data. Furthermore, the majority of studies identified for this review included participants who would be experiencing age-related declines in muscle mass and function [122]; we did not identify evidence from younger adults to enable the consideration of diet as an influence on the peak reached in mid-adulthood. Third, despite the smaller body of evidence from younger adult populations, the findings from dietary patterns studies already provide reasonably consistent messages about the role of diet quality; a growing number of studies show that higher quality diets are associated with beneficial effects on later physical performance. However, these are observational studies, and whole-diet intervention trials are urgently needed to understand this potential in different settings. Fourth, except for protein and vitamin D, there is relatively little evidence on links between intakes of individual nutrients in younger adult populations and subsequent muscle mass and function, which limits mechanistic understanding and the identification of specific nutrient targets for the prevention of sarcopenia. Finally, although there is widespread discussion about the need to increase protein intakes in later life, as well as a growing awareness of this among the general public [28], such beneficial effects of higher protein intakes are not supported by observational or trial evidence unless combined with exercise training.

Diets of higher quality, characterized by the frequent consumption of fruits, vegetables, wholegrain foods, legumes, and fish, but lower consumption of highly processed foods, are linked to a range of health outcomes, with substantial evidence showing greater compliance with healthier patterns of diet associated with a lower risk of noncommunicable diseases including cardiovascular disease, diabetes, cancer, and lower all-cause mortality [123]. The recent growth in dietary patterns studies indicates the potential of beneficial effects of higher quality diets for muscle function in older age, particularly linked to better-measured physical performance in older age [51,52]. The studies summarized in this narrative review show positive associations between diet quality in mid-adulthood and muscle outcomes assessed 10–20 y later. There is insufficient evidence to address whether these are causal influences, with the potential for cumulative effects with longer duration of exposure [78], or an indicator of the tracking of diet such that higher diet quality in mid-life predicts higher diet quality in older age. Both mechanisms are possible, although findings from the NSHD cohort, showing gains in physical performance in association with net improvements in diet quality at around retirement age, suggest that interventions to change diet in young-older age could have significant potential as an influence on sarcopenia risk [55]. However, further data from different settings are needed to understand the timing and possible cumulative effects of exposure to diets differing in quality for the prevention of sarcopenia.

Some of the most compelling evidence supporting the role of diet quality comes from the Baltimore Longitudinal Study of Aging, reported by Talegawkar et al. in 2022 [41]. Greater compliance with the MIND diet (combining elements of the Mediterranean and Dietary Approaches to Stop Hypertension Trial dietary patterns) was associated with better physical performance, and importantly, a slower rate of functional decline over time, with consistent findings among males and females. This dietary pattern emphasizes plant-based foods, specifically phytonutrient-rich foods (e.g., berries, green leafy vegetables), wholegrain foods, and fish, but limits the consumption of fast or fried foods, red meat and products, pastries, and sweets. Such healthier food profiles make up a nutrient-dense diet and would be expected to provide higher intakes of a range of nutrients linked to beneficial effects on muscle function, as well as having potential effects on gut microbiota that may be important [35,124,125]. Healthy eating messages are well-understood by the general public and interventions to improve diet quality are effective. However, the potential benefits for muscle function and physical performance may not be widely known. Although the combined benefits of exercise training with improved diet quality need to be established, evidence from protein supplementation studies suggest that such muscle health promotion strategies may be most effective when combined with messages to encourage physical activity.

Lifecourse studies show that differences in the pace of aging are evident early in adulthood [126], highlighting opportunities for health promotion from younger ages. As current evidence points to the need to promote diet quality and physical activity, possible muscle health promotion efforts would share common features with existing strategies for cardiovascular health promotion and cancer prevention, which should make them simpler to communicate. There are many gaps in current evidence on the role of diet in the etiology of sarcopenia, which need to be addressed in future research. However, there is also a case to communicate what is already known: that healthier diets of higher quality across adulthood, with known benefits for a number of health outcomes, are also linked to effective preservation of muscle mass and function - with the potential to enable better physical performance and independence in older age.

## Acknowledgments

The authors’ responsibilities are as follows – all authors: contributed to the design and concept of this review; SR: drafted the manuscript; all authors: critically reviewed the draft and provided detailed commentary and changes that were incorporated in its revision; and all authors: read and approved the final manuscript.

### Conflicts of interest

The authors report no conflicts of interest.

### Funding

The authors reported no funding received for this study.
